# Primary health care nurses’ mental health knowledge and attitudes towards patients and mental health care in a South African metropolitan municipality

**DOI:** 10.1186/s12912-023-01188-x

**Published:** 2023-01-28

**Authors:** Nanteza Gladys Kigozi-Male, James Christoffel Heunis, Michelle Catherine Engelbrecht

**Affiliations:** Centre for Health Systems Research & Development, University of the Free State, P.O. Box 339, Free State Province 9300 Bloemfontein, South Africa

**Keywords:** Mental health disorders, Primary health care, Nurses, Tuberculosis, Knowledge, Attitudes, South Africa, Integration

## Abstract

**Background:**

In South Africa, there are on-going calls to integrate mental health services into existing primary health care (PHC) programmes such as Tuberculosis (TB). Successful service integration and quality service delivery partially depend on healthcare providers’ mental health-related knowledge and attitudes. The aim of this study was to assess PHC nurses’ mental health knowledge and attitudes towards mental health patients and mental health care.

**Methods:**

This was a cross-sectional survey involving the distribution of self-administered questionnaires among PHC nurses across 47 clinics. Data on socio-demographics, stigma-related mental health knowledge, and nurses’ attitudes towards people with mental health problems and mental health care were subjected to descriptive and multiple regression analyses.

**Results:**

Out of 205 respondents, the majority were female (*n* = 178, 86.8%). The nurses’ median age was 50 (interquartile range: 39–56). Their mean mental health knowledge score was 23.0 (standard deviation [sd]: 3.07) out of 30. Nurses were less knowledgeable about the employment (*n* = 95, 46.3%), recovery (*n* = 112, 54.6%), and help-seeking behaviour (*n* = 119, 58.0%) of people with mental health problems. Professional nurses had a significantly higher mean mental health knowledge score than enrolled/assistant nurses (22.8 vs. 21.1, *t*_203_ = 4.775, *p* < 0.001). Regarding attitudes, the nurses’ mean attitude score was 40.68 (sd: 9.70) out of 96. Two in every five nurses (*n* = 91, 44.4%) scored above the mean attitude score, implying that they were inclined to have negative (stigmatising) attitudes towards people with mental problems and mental health care. Age (*p* = 0.048), job category (*p* < 0.001), and prior in-service mental health training (*p* = 0.029) made a unique contribution to predicting nurses’ attitudes.

**Conclusion:**

Gaps were established in PHC nurses’ stigma-related mental health knowledge. A significant proportion of nurses had a propensity for negative (stigmatising) attitudes towards mental health patients and mental health care. Efforts towards integration of mental health into TB services in this metropolitan and similar settings should address mental health knowledge deficits and factors influencing nurses’ negative attitudes. In-service training on mental health should be optimised, with attention to older nurses and enrolled/nursing assistants.

## Background

Mental health is increasingly recognised as a global priority owing to the negative impact of mental health disorders on populations. A systematic analysis of the global burden of disease highlighted that mental health disorders have consistently formed more than 14% of age-standardised years lived with disability for nearly three decades, with a global prevalence of more than 10% [1]. This has serious implications for resource-constrained settings like South Africa where the already fragile health system is struggling under a surging burden of communicable diseases such as Tuberculosis (TB). National surveillance data on mental health disorders in South Africa is scarce. A widely-cited 2009 population-based study reported that the 12-month prevalence of common mental health disorders among South African adults was 16.5% and the lifetime prevalence was 30.3% [2]. In 2012, another study [3] reported particularly high levels of psychological distress among South Africans aged 15 years and older, with an average national prevalence of 28.4%.

The Life Esidimeni debacle in the Gauteng Province of South Africa leading to a large number of patients dying prematurely due to neglect and poor service delivery [4] unveiled the human rights crisis facing mental health care in South Africa. Other international and national reports [5, 6] have also highlighted gaps in mental health care in the country. A Lancet National Commission consensus report on health care in South Africa [5] and a South African Human Rights Commission report on the status of mental health in South Africa [6] cited that the spate of mental health conditions is perpetuated by the non-prioritisation of mental health and under-investment in service delivery. This has further stimulated research and discussions on how to improve access to mental health care in South Africa [7–9].

There are growing calls for the integration of mental health services into other primary health care (PHC) [10–12] including the TB programme. Particularly, evidence of increased comorbidity between mental health disorders and TB [13, 14], with mental health rates of up to 84% reported among TB patients [13], has accentuated the necessity for integrated services. This would not only facilitate the timely diagnosis of mental health conditions among TB patients, but also the monitoring of potential interactions between the drugs used to treat mental health conditions and TB. However, it remains unclear how service integration can be successfully achieved under routine care conditions.

The *National Mental Health Policy Framework and Strategic Plan 2013–2020* [15] promulgated in 2013 provides guidance on mental health promotion, prevention of mental illness, treatment and rehabilitation for diverse mental health disorders in South Africa with limited focus on the integration of mental health into other PHC programmes such as TB. Among other factors, the successful integration of mental health services into the TB programme is contingent upon health workers' mental health knowledge and attitudes towards mental health care. The general expectation is for healthcare providers to be knowledgeable about mental health conditions and to be humane towards patients [16]. However, research on the African continent has revealed that healthcare providers often have inadequate mental health knowledge and tend to grapple with identifying and treating mental health conditions [17–23]. Studies have also reported a propensity for negative attitudes towards people with mental health problems and the provision of mental health services among healthcare providers [22–27]. Such limitations are bound to challenge health workers’ ability to provide good quality services and could aggravate patient treatment outcomes.

Despite their pivotal role in health service delivery, few studies in Africa have appraised PHC nurses’ mental health knowledge and attitudes towards mental health care. Available research has highlighted that nurses’ negative attitudes towards mental health patients and mental health care are linked to various personal factors including inadequate mental health knowledge, inadequate training, inexperience in mental health care, religious predisposition, low levels of education, fear related to the mental healthcare environment, and the lack of personal experience of mental illness [16, 18, 24–27]. As previous studies conducted in South Africa have concentrated on nurses working at general hospitals [25–30], the current study targeted PHC nurses with the goal to inform efforts to integrate mental health into TB services in a resource-constrained metropolitan in South Africa. The aim of this study was to assess PHC nurses’ mental health knowledge and attitudes towards mental health patients and mental health care.

## Methods

### Design and setting

The research followed a cross-sectional design using self-administered questionnaires distributed among PHC nurses working across 47 clinics in a metropolitan municipality in South Africa. The municipality has an estimated population of 787 803 [31], with about 84% of the uninsured population accessing public health care [32]. The municipality was purposively selected based on a high burden of disease and poor treatment outcomes [33, 34]. For instance, in 2018, the TB treatment success rate in this municipality was below the national 90% target, at 78.4% [34].

### Participants

The participants in this study were nurses working at public sector PHC facilities in a metropolitan municipality. All those working as nursing assistants, enrolled nurses, student nurses or professional nurses at any of the 47 public PHC facilities in the metropolitan municipality were eligible to participate in the study.

### Instrument and data gathering

A structured self-administered questionnaire was developed for data gathering. The questionnaire measured socio-demographic information, stigma-related mental health knowledge, and attitudes towards mental health care. Ten items measured socio-demographic attributes including sex, age, sub district, highest educational qualification, years of experience as a healthcare provider, job category, prior in-service training on mental health, self-reported screening of patients for mental health conditions, and self-reported referral of patients for mental health evaluation.

Mental health knowledge was assessed using six items from the Mental Health Knowledge Schedule (MAKS). The MAKS consists of 12 items, the first six items measure stigma-related mental health knowledge areas including help-seeking, recognition, support, employment, treatment and recovery. The last six items assess the levels of recognition and familiarity with clinical conditions including depression, stress, schizophrenia, bipolar disorder, drug addiction and grief [35]. As this study was more concerned with nurses’ stigma-related mental health knowledge and not necessarily diagnosing mental health conditions, only the first six items of the MAKS were considered in this study. Respondents were asked to indicate the extent to which they agreed or disagreed with the six statements. Items in which the respondent strongly agreed with the correct statement were accorded a value of 5. Statements in which the respondent strongly disagreed with the correct statement were accorded a value of 1. The MAKS has moderate internal consistency among the six items, with a Cronbach alpha of 0.65 [35]. The authors declared that the MAKS was not designed to function as a scale; it contains heterogeneous items measuring multi-dimensional aspects of stigma-related mental health knowledge. Despite fair reliability, the MAKS has received positive rating for its content validity [36].

Nurses’ attitudes towards people with mental health problems and mental health care were assessed using the Mental Illness Clinicians’ Attitudes scale (MICA version 4) [37]. This scale is an extension of the MICA version 2 [38] and is designed to assess the attitudes of any clinical healthcare provider toward people with mental health problems as well as mental health care. Responses were scored on a 6-point Likert scale as follows: strongly disagree = 6, disagree = 5, somewhat disagree = 4, somewhat agree = 3, agree = 2, and strongly agree = 1. Items 1, 2, 4, 5, 6, 7, 8, 13, 14 and 15 were reverse scored such that strongly agree = 6, agree = 5, somewhat agree = 4, somewhat disagree = 3, disagree = 2, and strongly disagree = 1. The MICA v4 has been reported to have satisfactory internal consistency with a Cronbach alpha value of 0.7 and is reliable for use among health professionals such as nurses [19, 37]. In the current study, assessment of the internal consistency of the MICA-4 scale yielded a Cronbach alpha of 0.7, implying that the scale was reliable to use among PHC nurses in our setting.

Data were gathered from October to November 2020. A trained fieldworker dropped off questionnaires and envelopes together with information about the study at the PHC facilities. With the assistance of facility managers, all the nurses, including professional, enrolled or nursing assistants working at the facilities were invited to complete the questionnaires. Respondents were informed that by completing the questionnaires they were voluntarily agreeing to participate in the study. Completed questionnaires were enclosed in sealed envelopes and collected within two weeks of being dropped off at the PHC facilities.

### Data analysis

Data were double-captured, cleaned and analysed using SPSS, version 27 [39]. Regarding mental health knowledge, response values for each item were summed up to generate a total score, with higher scores reflecting a better stigma-related mental health knowledge. Similarly, mental health attitude scores for each item were summed to produce a single attitude score. A high overall score reflected negative (stigmatising) attitudes towards mental health patients and mental health care. T-tests were performed to assess differences in nurses’ mean knowledge scores. Multiple linear regression analysis was used to establish the determinants of nurses’ attitudes towards mental health patients and health care at a statistical significance level of *p* ≤ 0.05. Preliminary analyses were performed to ensure that the assumptions of linearity, independence of errors, homoscedasticity, unusual points and normality of residuals were met.

## Results

### Socio-demographic characteristics

Out of 260 questionnaires distributed, 205 completed questionnaires were returned, resulting in a response rate of 78.9%. Table 1 depicts the respondents’ socio-demographic information. A large proportion of the sample were female (*n* = 178, 86.8%). Respondents’ median age was 50 (interquartile range: 39–56). Just over half (*n* = 112, 54.6%) of the nurses had attained a nursing college qualification. A slight majority of the sample was working in clinics located in small towns (*n* = 119, 58.0) in the municipality. The mean duration of employment as a healthcare provider was 16.6 (sd: 11.7) years. Two-thirds of the respondents were professional nurses (*n* = 137, 66.8%). Most of the nurses (*n* = 121, 59.0%) indicated that they had not received prior in-service training on mental health, however, more than three-quarters claimed to have screened patients for mental health conditions (*n* = 160, 78.0%) and referred (*n* = 156, 76.1%) those with suspected mental health problems for further evaluation. The nurses’ mean stigma-related mental health knowledge score was 23.0 (sd: 3.07) out of 30, two in every ten nurses scored below the mean implying that they were less knowledgeable. The nurses’ mean attitude score was 40.68 (sd: 9.70) out of 96, two in every five nurses scored above the mean score, implying that they were inclined to have negative (stigmatising) attitudes towards people with mental health problems and mental health care.

**Table 1:** Socio-demographic information (*N* = 205)

Variable	n (%)
**Sex**	
Male	27 (13.2)
Female	178 (86.8)
**Age groups [years] (median (IQR)**^a^	50 (39–56)
**Highest education qualification**	
Matric (Grade 12)	10 (4.9)
Nursing college	112 (54.6)
University	83 (40.5)
**Clinic location**	
City	86 (42.0)
Small town	119 (58.0)
**Duration of working as a health worker in years (mean, sd)**^b^	16.6 (11.7)
**Job category**	
Professional nurse	137 (66.8)
Enrolled nurse/nursing assistant	68 (33.2)
**Mental health knowledge (mean; sd)**	23.0 (3.07)
**Ever received in-service training on mental health?**	
Yes	84 (41.0)
No	121 (59.0)
**Do you screen patients for mental health conditions?**	
Yes	160 (78.0)
No	45 (22.0)
**Do you refer patients for mental health care?**	
Yes	156 (76.1)
No	49 (23.9)
**Attitudes towards people with mental health problems/mental health care (mean; sd)**	40.68 (9.70)

^a^Mean (sd) age: 46.59 (10.71) years

^b^Median (IQR) duration: 14 (6–27) years

Table 2 depicts nurses’ stigma-related mental health knowledge. Respondents were less knowledgeable about the employment, recovery, and help-seeking behaviour of people with mental health problems. Less than half (*n* = 95, 46.3%) of the respondents indicated that “*Most people with mental health problems want to have paid employment*.” Just over half of the respondents indicated that “*People with mental health problems can fully recover*” (*n* = 112, 54.6%), and “*Most people with mental health problems go to a healthcare professional to get help*” (*n* = 119, 58.0%). Further analysis established that professional nurses had a statistically significantly higher mean mental health knowledge score than enrolled/assistant nurses (22.8 vs. 21.1, *t*_203_ = 4.775, *p* < 0.001). The mean mental health knowledge score of nurses working in city-based clinics was also statistically significantly higher than the mean score of nurses working in smalltown- based clinics (22.7 vs. 21.8, *t*_203_ = 2.632, *p* = 0.009). Furthermore, statistically significantly higher mean mental health knowledge scores were established for nurses who self-reported screening of patients for mental health conditions compared to those who did not (22.5 vs. 21.1, *t*_203_ = 3.384, *p* = 0.001), and nurses who self-reported referring patients for further mental health evaluation compared to those who did not (22.4 vs. 21.3, *t*_203_ = 2.759, *p* = 0.006).

**Table 2:** Nurses’ stigma-related mental health knowledge

Statement	Strongly agree/agree
	**n (%)**
Most people with mental health problems want to have paid employment (true)	95 (46.3)
If a friend had a mental health problem, I know what advice to give them to get professional help (true)	190 (92.5)
Medication can be an effective treatment for people with mental health problems (true)	182 (88.8)
Psychotherapy (e.g. talking therapy or counselling) can be an effective treatment for people with mental health problems (true)	186 (90.7)
People with severe mental health problems can fully recover (true)	112 (54.6)
Most people with mental health problems go to a healthcare professional to get help (false)	119 (58.0)

### Nurses’ attitudes towards mental health patients and mental health care

Results of the multiple linear regression to determine the predictors of nurse attitudes towards patients and mental health care are depicted in Table 3. Multiple linear regression analysis was performed to investigate the relationship between nurses’ sex, age, geolocality, work experience, job category, mental health knowledge, prior mental health in-service training, patient referral for mental health care, and their attitudes towards mental health patients and mental health care. The inclusion of these variables into the model was informed by previous research on factors associated with healthcare providers’ attitudes to mental health care [24–30, 40–42]. To achieve 80% statistical power with eight predictor variables, and a medium (0.15) effect size, a minimum sample size of 109 was required for the multiple regression analysis. The assumptions of linearity, independence of errors, homoscedasticity, unusual points and normality of residuals were met. The scatterplot of standardised predicted values versus standardised residuals showed that the data met the assumptions of homogeneity of variance and linearity. Also, the residuals were approximately normally distributed and there were no unusual points in the data. The aforementioned independent variables together statistically significantly predicted nurses’ attitudes (F_[8,204]_ = 6.927, *p* < 0.001, adjusted *R*^2^ value = 0.189). However, only age (*p* = 0.048), job category (*p* < 0.001), and prior in-service mental health training (*p* = 0.029) made a unique contribution to this prediction. For age, there was a 0.186 increase in nurses’ attitudes scores with every unit increase in nurses’ age. Enrolled nurses’ attitude scores were 7.745 times higher than professional nurses’ scores, and nurses who had not received prior in-service training on mental health scored 3.219 times higher than their counterparts who had attended such training.

**Table 3:** Determinants of nurses’ attitudes towards mental health patients and mental health care

Variable	Unstandardised coefficients		*p*-value
	**B**	**Standard error**	
**Sex**	-0.596	1.842	0.747
Male (ref)			
Female			
**Age**	0.186	0.093	0.048
**Job experience**	0.040	0.090	0.656
**Subdistrict**	0.662	1.306	0.613
Urban (ref)			
Rural			
**Job category**	7.745	1.652	< 0.001
Professional nurse (ref)			
Enrolled/assistant nurse			
**Mental health knowledge score**	0.154	0.208	0.459
**Prior mental health in-service training**	3.219	1.468	0.029
Yes (ref)			
No			
**Referred patients for mental health care**	-0.420	1.799	0.816
Yes (ref)			
No			
**Constant**	21.364	7.652	0.006

Overall *p* < 0.001; *R*^2^ = 0.220; adjusted *R*^2^ = 0.189; *ref* = Reference

## Discussion

This study examined PHC nurses’ stigma mental health-related knowledge and attitudes towards mental health patients and mental health care. Similar to reports in other African studies that identified gaps in mental health knowledge among non-specialist PHC providers [21, 40, 41], a substantial proportion of nurses in this study seemed to lack knowledge about employment, recovery and help-seeking behaviour of people with mental health problems. Significantly lower levels of mental health knowledge were established among enrolled/assistant nurses compared to professional nurses, those working in small town-based clinics compared to those working in city-based clinics, nurses who self-reported not to have screened patients for mental health conditions compared to those who did, and nurses who self-reported not to have referred patients for mental health evaluation compared to those who did. While nurses are generally expected to have better mental health knowledge than the general population, nurses may lack specific knowledge which may potentially negatively affect their healthcare service delivery, by for instance, engendering discrimination of patients, which in turn may limit help-seeking among patients. This suggests a need for mental health awareness and (refresher) training for the nurses. In light of the ongoing calls in South Africa for the integration of mental health services into other PHC programmes such as the TB programme, there is a further need to develop appropriate strategies for the implementation of mental health interventions among non-specialist nurses. In Nigeria, a demonstration project used the mental health gap action programme intervention guide involving the training of master trainers to train facilitators who would later train front-line PHC workers on various aspects of mental health care. This training model was reported to be pragmatic and cost-effective for enhancing mental health care in settings with a scarcity of mental health specialists [43].

In terms of nurses’ attitudes, in line with an Ethiopian study reporting that nearly 50% of PHC nurses harboured negative attitudes towards people with mental health problems [19], two in every five nurses who completed the self-administered questionnaire were inclined to have negative (stigmatising) attitudes towards people with mental health problems and mental health care. Age, job category and in-service training made a significant contribution towards predicting nurses’ attitudes to patients and mental health care. Based on the current findings, older nurses, enrolled/nursing assistants were inclined to harbour negative (stigmatising) attitudes. A systematic review of studies conducted in developed and developing countries also alluded to the preponderance of negative attitudes among healthcare providers, which would benefit from behaviour change interventions [24]. Consequently, efforts to integrate mental health services into the TB programme should consider (evidence-based) interventions [23, 42] to address nurses’ misconceptions about mental health disorders and promote positive attitudes towards patients and mental health care. According to de Jacq and colleagues [44], implementing policy guidelines for the management of diverse mental health conditions could foster a sense of empowerment among nurses, thereby promoting a more positive outlook on mental health disorders. Management could also play an influential role by overtly promoting a culture of tolerance towards mental health patients and mental health care.

Besides personal characteristics, current findings indicate that lack of in-service training on mental health care may engender negative (stigmatising) attitudes among nurses towards mental health patients and mental health care. Research among medical officers and professional nurses in South Africa found that brief training helped to boost participants’ confidence in self-reported competency and referral behaviour; trainees were more confident in performing mental healthcare-related activities and managing mental health conditions, which contributed to a decrease in the number of upward referrals over the study period [45].

A limitation of the current study is that the nurses’ knowledge and attitudes towards people with mental health problems could not be linked to their actual behaviour. Thus, efforts towards the integration of mental health services into the TB programme should explore nurses’ needs and also report on their actual mental health-related practices. Qualitative research could also provide further insights into nurses’ views and perceptions of mental health patients and mental health care. It was also not possible to compare nurses’ knowledge and attitudes to the general population accessing PHC services. Furthermore, the data were collected from a convenient sample of PHC nurses, which limits the generalisability of the research findings. The sample size provided sufficient statistical power to perform the multiple regression analysis, however, the explanatory value of the regression model was low. While the internal consistency of the MICA-4 was satisfactory, implying that the scale was reliable to use in our study setting, future research should verify the five-factor latent structure underlying the 16-items as suggested by Gabbidon and colleagues. Nonetheless, the findings provide good enough understanding of the factors contributing towards nurses’ attitudes towards mental health patients and mental health care in a resource- constrained metropolitan PHC setting.

## Conclusion

The findings help to address the dearth of literature on mental health knowledge and attitudes towards mental health patients and mental health care among non-specialist nurses in PHC settings in South Africa. The study established gaps in PHC nurses’ stigma-related mental health knowledge. At the same time, there was a propensity for negative (stigmatising) attitudes towards mental health patients and mental health care. In light of ongoing calls for the integration of mental health services into other PHC programmes such as TB, it is imperative to explore nurses’ mental health awareness and address the factors influencing negative attitudes towards mental health patients and mental health care. Specific attention should be given to older nurses and enrolled/nursing assistants. Programme integration efforts should optimise in-service training on mental health for non-specialist nurses to facilitate quality service delivery.

## Data Availability

The datasets used and/or analysed during the current study are available from the corresponding author upon reasonable request.

## Abbreviations

PHC: Primary health care
TB: Tuberculosis
